# Embracing additive manufacture: implications for foot and ankle orthosis design

**DOI:** 10.1186/1471-2474-13-84

**Published:** 2012-05-29

**Authors:** Scott Telfer, Jari Pallari, Javier Munguia, Kenny Dalgarno, Martin McGeough, Jim Woodburn

**Affiliations:** 1 School of Health and Life Sciences, Glasgow Caledonian University, Cowcaddens Road, Glasgow, UK; 2 Peacocks Medical Group Ltd, Benfield Business Park, Newcastle, UK; 3 School of Mechanical and Systems Engineering, Newcastle University, Claremont Road, Newcastle, UK; 4 Firefly Orthoses Ltd, Markievicz Road, Sligo, Ireland

**Keywords:** Additive manufacture, 3D printing, Foot orthoses, Ankle-foot orthoses, Biomechanics

## Abstract

**Background:**

The design of foot and ankle orthoses is currently limited by the methods used to fabricate the devices, particularly in terms of geometric freedom and potential to include innovative new features. Additive manufacturing (AM) technologies, where objects are constructed via a series of sub-millimetre layers of a substrate material, may present the opportunity to overcome these limitations and allow novel devices to be produced that are highly personalised for the individual, both in terms of fit and functionality.

Two novel devices, a foot orthosis (FO) designed to include adjustable elements to relieve pressure at the metatarsal heads, and an ankle foot orthosis (AFO) designed to have adjustable stiffness levels in the sagittal plane, were developed and fabricated using AM. The devices were then tested on a healthy participant to determine if the intended biomechanical modes of action were achieved.

**Results:**

The adjustable, pressure relieving FO was found to be able to significantly reduce pressure under the targeted metatarsal heads. The AFO was shown to have distinct effects on ankle kinematics which could be varied by adjusting the stiffness level of the device.

**Conclusions:**

The results presented here demonstrate the potential design freedom made available by AM, and suggest that it may allow novel personalised orthotic devices to be produced which are beyond the current state of the art.

## Background

Currently, the design of custom and customised orthoses for the foot and ankle is heavily restricted by the materials and methods used to fabricate the device. Perhaps the most common approach involves vacuum forming a thermoplastic sheet around a balanced, corrected positive plaster cast of the anatomy of interest, then cutting away unwanted material to form the orthosis [1,2]. Some manufacturers may also utilise a standardised range of moulds of varying size and shape that can be chosen based on a few predefined measurements from the patient, however the basic fabrication process remains the same [3].

Manufacturing devices in this way provides limited scope for the incorporation of innovative features requiring alterations to the form of the device. Recently, the ability to digitise parts of the anatomy directly or from impression casts has meant that computer aided design and manufacturing (CAD/CAM) tools can be used to create the orthosis shape. As a result, direct milled custom devices where the orthosis is carved out of a solid piece of material have gained in popularity [4]. However, again the ability to incorporate truly novel features using this approach is still limited due to the nature of the manufacturing method.

Additive manufacturing (AM), also commonly known as 3D printing, rapid prototyping or solid freeform manufacture [5], is a technology which utilises layer manufacturing and has the ability to surmount these limitations and allow healthcare professionals involved in the prescription of these types of devices the opportunity to explore truly novel orthotic design features.

AM has existed for two decades, however the initial investment involved in machine and ancillary equipment acquisition and the restrictions in terms of mechanical properties of the available materials has generally constrained its use primarily to small scale prototyping within a few specific industries. Recently however, technological advances and moves towards a mass customisation business model have meant that the cost and expertise required to exploit AM have decreased significantly [6]. Some predictions have been made suggesting that in the future 3D printers may become as ubiquitous in our homes and offices as 2D printers are today [7] and there are already a number of relatively low cost (~£2K or less) systems available, with drivers in place to reduce these hardware costs further [8]. While these lower cost machines are primarily suitable only for low volume manufacturing purposes, they demonstrate that the technology is no longer an esoteric tool limited to highly specific applications. A number of systems are able to produce parts in high strength and durability engineering plastics such as polypropylene and acrylonitrile butadiene styrene (ABS), and even metals such as titanium, stainless steel and various ranges of alloys [6], meaning that it is possible to manufacture fully functional components suitable for load bearing use.

Recently, a number of papers have been published presenting foot orthoses (FOs) and ankle foot orthoses (AFOs) fabricated using AM techniques, successfully demonstrating the feasibility of this approach [9-13]. However, it is worth noting that these studies have tended to use designs similar to those produced using traditional methods, rather than fully exploit the design freedom provided by the technology.

This article provides a brief overview of AM technology with reference to ankle and foot orthosis fabrication. Pre-clinical testing results for two prototype designs are presented, and these concepts are intended to illustrate the potential of AM to allow innovative new designs to be developed and provide a greater range of prescription options for clinicians.

### AM techniques

Additive manufacture is an umbrella term which covers a range of technologies that utilise layer manufacturing to fabricate items. These items originate as a 3D computer model, usually in the .stl format, which is converted into a code containing instructions for the manufacturing machine which will fabricate the item. There are many variations of AM but the three main approaches will be covered here: selective laser sintering (SLS), stereolithography (SLA), and fused deposition modelling (FDM).

**Figure 1:**
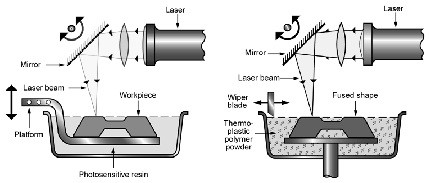
Process schematic for SLA (left) and SLS (right).

Rather than carving the desired object out of a solid block of material as is the case in direct milling, in all AM approaches the desired object is built of sub-millimetre thick layers of a substrate material (SLA and SLS), or of a directly extruded build material (FDM). For techniques using a substrate, the material is laid out as a thin, uniform layer of liquid resin (SLA) or powder (SLS) covering the build area (Figure 1). A laser beam then traces out the cross sectional shape of the item being built in the substrate and this cures (SLA) or sinters (SLS) the area of interest into a solid. The build platform is then lowered (typically between 0.05 and 0.2mm depending on the accuracy required) and another layer of substrate material laid down, with this process being repeated for the required number of layers until the item is built.

**Figure 2:**
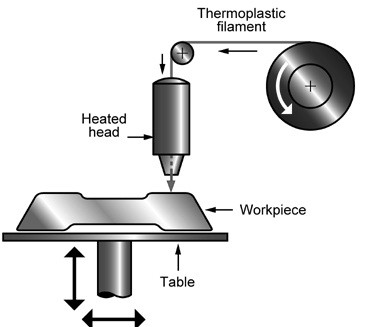
Process schematic for FDM process.

With FDM, the material the item is to be built out of (normally a thermoplastic) is fused and extruded as a thin line and the build platform and/or the extruder itself is moved so that the cross sectional shape of the item is produced (Figure 2). To save time and material, usually the outline of the shape is printed and the enclosed area filled with a honeycomb or other pattern chosen by the operator, depending on the strength/build speed requirements of the item. Again, the build platform is lowered and the next layer printed on top of the preceding one until the item is complete. Support structures may also need to be included to allow overhanging parts of the item to be built.

**Figure 3:**
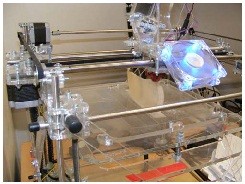
**Foot orthosis fabrication**. FO being printed in polylactide (PLA) on a low cost FDM machine (RapMan; Bits from Bytes, Clevedon, UK).

As the cost range of industrial AM systems goes from €15k to €500k depending on the capacity, build size and material used, a number of open-source and low cost initiatives have recently emerged. Although at the moment limited by overall precision and repeatability issues, some low cost systems (€1k to €3k) based on FDM have consolidated themselves as firm candidates for on-site manufacturing (Figure 3).

These fabrication methods make it possible to manufacture detailed, geometrically complex objects requiring sub-millimetre resolution with relative ease. This is one of the primary reasons that AM is appealing for foot and ankle orthotic manufacture, where complex surface anatomy, potentially including deformities, is regularly encountered and needs to be accommodated. One of the major appeals of AM is that the cost of manufacturing a part tends not to increase with the complexity of the part, only with its volume. Additionally, due to the nature of SLS and SLA, the build time per device decreases significantly as the number of devices being fabricated in each “run” of the machine increases, making devices suitable for mass customisation an ideal candidate for these technologies.

## Methods

To demonstrate the potential of this approach we present two prototype devices which exploit the design freedom provided by AM. It should be stressed that these are prototype designs to illustrate proof-of-principle and have not been tested in patient populations.

### FO with adjustable metatarsal support elements

**Figure 4:**
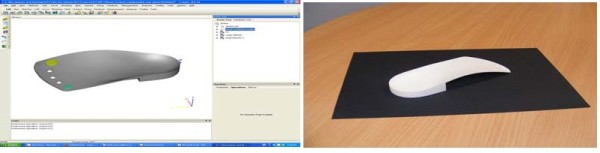
**FO with adjustable metatarsal support elements.** CAD model (left) and fabricated device (right). 2^nd^ to 4^th^ adjusters not shown in CAD model for clarity. Sections of the adjustable elements and their corresponding holes in the FO are threaded, allowing their height to be easily adjusted with a screwdriver.

Forefoot pain at the metatarsal heads can often be relived by reducing the loading on one or more of the distal metatarsal head using an FO modification known as a metatarsal bar or dome [14]. This modification is intended to redistribute a proportion of the load away from the metatarsal head and onto a more proximal area of the foot. This feature can be added as an intrinsic part of the device at the design stage (in the case of direct milled orthoses) or more commonly as additional material which is attached to an existing device. The design presented here (Figure 4) includes a number of areas under the metatarsals which can be individually raised to different heights, from approximately 0.5mm to 3mm, above the surface of the device. The adjustable elements and their corresponding holes in the FO are threaded, allowing easy adjustment of height using a screwdriver. The intention with this design is to provide the clinician with the ability to quickly and easily trial a number of permutations to maximise pain and/or pressure relief at the metatarsal heads without the need to add or remove material from the device.

The FO device is based on a custom three-quarter length orthosis CAD model, designed from a direct scan of the particant’s foot and exported in .stl format from OrthoModel (Delcam Ltd, Birmingham, UK), a commercially available FO software design package. Modifications to the design were made in 3-matic (Materialise NV, Leuven, Belgium) and the device manufactured using an EOSINT P 700 SLS machine (EOS GmbH, Munich, Germany) in PA2200 Nylon-12 powder, also from EOS, by Materialise NV.

### Adjustable stiffness AFO

AFOs are prescribed to improve pathological gait in patients with muscular strength and/or control problems around the ankle. It has been suggested that an optimal match exists between the stiffness or rigidity of the device and the patient [15]. Additionally, our experience suggests that the ability to adjust the sagittal plane stiffness of an AFO may have benefits in terms of allowing the user to tailor the functional performance of the device to the activity they wish to perform. For example, a very rigid AFO may help maximise efficiency during flat walking, however the patient may prefer a less rigid device for ascending and descending stairs.

**Figure 5:**
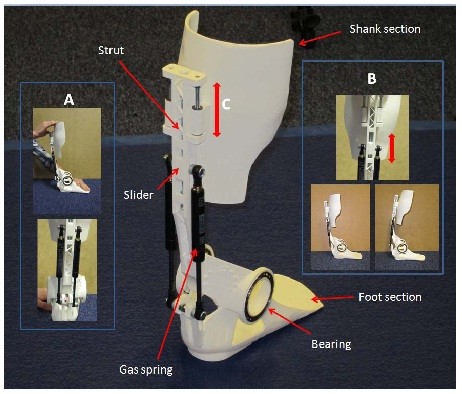
**Adjustable stiffness ankle foot orthosis. A**) In the lower stiffness condition, when the gas spring on the medial side compresses to provide resistance to plantarflexion, the disengaged spring on the lateral side is free to slide down its support bracket without giving any resistance. **B**) The slider component provides the upper attachment point of the gas springs and is held in place by two M6 bolts (one above and one below). By adjusting these bolts the slider can be moved up and down, and this alters the shank to foot angle. The adjustment range is approximately 6° of anterior and posterior tilt. **C**) The shank section is mounted on runners to allow it to move up and down freely. This is intended to reduce friction between the calf and this component of the device during gait.

The design presented here is essentially a dynamic AFO and consists of four AM components: shank section, strut, foot section, and slider (Figure 5). Additionally, off-the-shelf components consisting of two bearings, two gas springs and a number of nuts, bolts and washers are used.

As well as the AM components demonstrating the geometric freedom of the manufacturing process, the design has three features not commonly included in traditional AFO designs-

A. The two adjustable gas springs are attached to the posterior side of the AFO to give resistance plantarflexion. The gas spring on the medial side can be quickly disengaged from its attachment point on the lower bracket **via a simple mechanism, the inclusion of which was made possible by AM,** meaning that the sagittal plane stiffness is provided only by the gas spring on the lateral side. This allows the device to be set to provide two different levels of stiffness, **each potentially suitable for different activities, and for the user to quickly switch between the two settings**.

B. The **strut features and intricate design allowing the** attachment point for the gas springs **to be** moved up and down, and as a result this means the shank to foot angle can be altered **in a quick and simple manner, potentially advantageous for testing various angle during a clinical assessment to maximise benefit to gait**.

C. The shank section is able to slide up and down two runners at the top of the strut, compensating for any friction generated during plantar flexion by misalignment of the hinge axis of the device and the ankle.

The design for the AFO device was based around a 3D surface scan of a plaster cast of the lower limb of the test subject, with the CAD model developed using 3-matic software and manufactured by Materialise NV using the same material and equipment as the FO described in the previous section. The form of the shank and foot sections are anatomically based around the scan of the cast, allowing a custom fit of the parts directly in contact with the leg and foot to be achieved. These parts were then modified to include the necessary attachment points for the remaining AM and off the shelf components.

A single participant (male, 29 years, weight 78kg, height 1.85m) tested both devices and provided informed consent before data collection began. All experimentation took place at Glasgow Caledonian University’s motion analysis laboratory and ethical approval was granted by the institutional ethics committee. The participant’s natural self selected walking speed was determined prior to the measurements and metronome and timing gaits used to ensure the walking trials did not exceed ±5% of the self selected speed.

### FO testing

To test if the FO device had the intended biomechanical effects, an in-shoe pressure measurement system (Pedar-X; Novel Gmbh, Munich, Germany) was used to determine the loading during gait on the plantar surface of the foot. The insoles contain 99 capacitive cells distributed across the sensing area. Pressure measurements were recorded at 50Hz. The participant walked for three minutes each in two FO conditions: a) with the adjustable elements all at their lowest position (*i.e.* almost flush against the surface of the FO); and b) with the adjustable elements under the second and third metatarsals raised approximately 2mm above the surface, a level that was found to be comfortable for the participant. The hypothesis was that the peak pressure under the second and third metatarsal heads during walking would be lower in condition b).

### AFO testing

To test the biomechanical effects of the AFO device, the participant underwent three dimensional gait analysis. Kinematic and kinetic data were acquired using a 12 camera Oqus motion camera system (Qualisys AB, Gothenburg, Sweden) and a force plate embedded into the walkway (9286B; Kistler Instrument Corp, Amherst, NY). Clusters of four retroreflective markers were attached to the distal part of the thigh and shank, individual markers to the posterior and anterior iliac spines and greater trochanters, and shoe mounted markers on the heel and approximately over the 1^st^ and 5^th^ met heads. The shank cluster was positioned anteriorly to ensure that the AFO did not interfere with its positioning during gait. Ankle and knee joint centres were defined as 50% of the distance between additional markers placed over the medial and lateral malleoli, and medial and lateral epicondyles respectively. These additional markers were removed after the initial static trial.

Prior to the measurements, the AFO was adjusted so that the shank to foot angle was 90°. The stiffness of the AFO, as controlled by the pressure in the gas springs, was set such that no compression of either gas spring was seen during visual observation of the participant’s gait while both springs were engaged. For the second stiffness condition where only the medial spring is engaged, the pressure in this spring was reduced iteratively until approximately 20mm of compression was seen during gait. For each test condition, the participant was instructed to walk along the walkway until ten successful trials were captured. A successful trial was defined as the leg wearing the orthosis striking the force plate cleanly as part of an uninterrupted gait pattern. Three conditions were tested in total: shod only, and wearing the AFO at the two stiffness levels. It was hypothesised that there would be changes in the measured biomechanical variables in response to the altered stiffness of the device and against the shod only condition.

### Data analysis

For the FO testing, twelve steps were analysed for each condition using Automask software (Novel Gmbh, Munich, Germany). A modified version of the mask reported in Ramanathan et al. [16] was used, allowing the pressure under the individual metatarsal heads to be determined. Data were checked for normality (Shapiro-Wilk test) and means compared using a *t*-test or nonparametric equivalent. Bonferroni correction was applied to account for multiple comparisons, resulting in an α value of 0.01.

Movement files for the AFO testing were processed using Visual 3D software (C-Motion Inc, Germantown, MD). The variables of interest were: sagittal plane ankle angle and internal moment, and the sagittal plane knee angle and internal moment. Moments were anatomically referenced to the proximal segment and all analysis was for the stance phase of gait. One way analysis of variance followed by post hoc comparisons using Tukey’s test were performed for the following discrete variables: peak plantarflexion during the first 50% of stance; plantarflexion angle at the end of stance; peak ankle internal plantarflexion moment; peak knee flexion during the first 50% of stance; and peak knee internal flexion moment for the first 50% of stance.

## Results

### Fabrication of devices

The estimated time to manufacture the pair of FOs was 5 hours 33 minutes (based on three pairs being manufactured in the build) and the estimated total cost of the pair was €56. For the AFO components the estimated build time was 13 hours 13 minutes (based on one device being manufactured) and the estimated total cost was €461. The off-the-shelf components for the AFO cost an additional €73. Costs for the AM parts are the commercial prices provided by 3D printing service iMaterialise (Materialise NV, Leuven, Belgium) and exclude tax and shipping.

AM components were checked for dimensional accuracy and found to be within 0.1mm of the CAD model for all tested dimensions. The overall time to assemble the AFO was around 10 minutes, and the FO < 1 minute.

### FO with adjustable metatarsal elements

Peak pressures under all metatarsal heads for both conditions are given in Table 1. By raising the adjusters, peak pressures were significantly reduced by 22.9kPa and 12kPa under the 2^nd^ and 3^rd^ metatarsal heads respectively (p < 0.001 and p = 0.007). Additionally, there was a relatively large non significant reduction in peak pressure under the first metatarsal head of 21.9kPa.

**Table 1:** Peak plantar pressure at metatarsal heads (kPa)

			**PPP (SD)**		
	1^st^ MTH	2^nd^ MTH	3^rd^ MTH	4^th^ MTH	5^th^ MTH
Adjusters lowered	189.4 (14.2)	175.6 (12.3)	138.9 (9.9)	124.6 (11.5)	97.3 (15.2)
Adjusters raised	167.5 (26.7)	152.7 (13.4)	126.9 (9.9)	133.8 (32.5)	100.8 (27.8)
P-value	0.025	<0.001*	0.007*	0.368	0.702

*PPP* peak plantar pressure; *SD* Standard deviation; *MTH* metatarsal head.

* Statistically significant difference.

### Adjustable stiffness AFO

**Figure 6:**
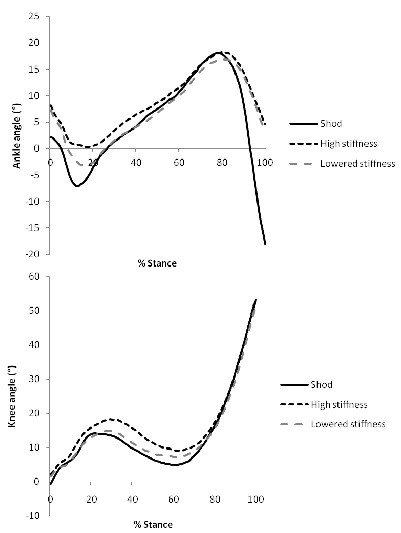
**Kinematics.** Mean ankle and knee kinematics in the sagittal plane for normal (shod) walking and high and low stiffness AFO conditions. Positive angles indicate (dorsi)flexion in the sagittal plane.

**Figure 7:**
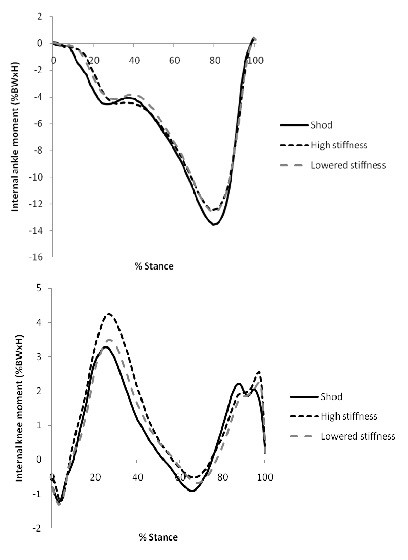
**Kinetics.** Ankle and knee kinetics in the sagittal plane for normal (shod) walking and high and low stiffness AFO conditions. Positive angles indicate: an internal dorsiflexion moment at the ankle; and an internal extension moment at the knee. %BWxH: percentage of the participant’s bodyweight multiplied by their height.

Motion and moment curves for the ankle and knee are presented in Figures 6 and 7 respectively. For ankle kinematics, significant differences were seen between all conditions for peak plantar flexion angle at the start of stance (p < 0.001) with the high stiffness setting allowing minimal flexion, followed by the lower stiffness setting, then the shod only condition. There was no difference between plantar flexion angle at toe off between AFO conditions (p = 0.336), however both were significantly lower in comparison to the shod condition (p <0.001). At the knee, there were significant differences between the high stiffness condition and both other conditions for peak flexion during the first half of stance (p < 0.001).

Peak ankle internal plantar flexion moment was significantly reduced in both AFO conditions compared to shod (p < 0.001), and both AFO conditions also increased peak knee internal flexion moment (p < 0.001) during the first half of stance (Figure 7).

## Discussion

In this article AM technology has been discussed with reference to its potential to be applied to the manufacture of customised ankle and ankle foot orthoses. Two novel devices have been presented, and results from short term pre-clinical tests provide preliminary evidence for their ability to cause the intended biomechanical mode of action in gait for a normal subject.

Inclusion of the novel features included in these designs requires the geometric freedom provided by AM to be fully exploited. In particular, the strut section of the AFO has an intricate geometry to allow the adjustment of the foot to shank angle and other attachment points while maintaining the strength required to withstand the forces generated during gait. This would be difficult to recreate using traditional manufacturing methods. The relatively simple placement of function elements relative to anatomical landmarks is another potential advantage enabled by AM that is demonstrated by the designs presented here.

For the FO design, the uncorrected values for peak metatarsal pressure was similar range to those previously reported in normal subjects using the same measurement system [16]. The reduction in pressure achieved using the adjustable elements was similar to that achieved and considered clinically relevant in a patient population [17] suggesting that clinical testing of the design may be warranted. However, the non significant reduction in the pressure under the first metatarsal head and increases in pressure under the fourth and fifth are possibly a result of the raised adjusters preventing full pronation of the forefoot during loading and this would need to be investigated further in a larger study group prior to testing this type of device in a clinical population.

In the case of the AFO design, the results here present preliminary evidence of the device’s ability to exert different biomechanical effects on the kinematics of the ankle in a normal subject. Significantly reduced and different levels of plantarflexion were seen between stiffness conditions during early stance phase, suggesting that it may be possible to use this type of device to allow patients to tailor the support provided to suit different activities, and this may be worth further investigation and optimisation of the design in the future. A study testing AFO designs in normal subjects also showed reductions at these points, and similar findings have been presented for post-stroke [18] and cerebral palsy populations [19]. The plantarflexion reduction at toe off also suggests that the device may provide the mechanical support necessary to control foot-drop during swing phase and reduce this risk of tripping, which is a common reason for prescribing an AFO [20].

In this study the stiffness of the device was set simply through observation of the participant’s gait while wearing the device, similar to the approach taken in current clinical practice where the trim lines of a polypropylene device may be altered to reduce the overall stiffness. Attempts are being made to develop standardised approaches for determining AFO stiffness [21], and since AM devices begin as a 3D computer model the opportunity exists to use computational modelling techniques such as finite element analysis to determine and potentially optimise the stiffness of the device prior to manufacture.

This study supports the findings of previously reported investigations of AM for orthotic design. A feasibility and material benchmarking study was carried out by Faustini et al. [9] into SLS fabrication of AFOs. They found that an SLS fabrication-based design analysis and manufacturing framework was “ideally suited for this application”. Three SLS materials were used to make AFOs, based on a commercially available carbon fibre AFO. Benchmarking exercises were undertaken in the form of evaluation of energy dissipation characteristics, rotational stiffness, and destructive testing with these values being compared against those of the existing device, and the most suitable material identified.

The feasibility of the SLS approach for manufacturing AFOs was replicated recently by Mavroidis et al. [10], who produced a personalised device which they then tested on a healthy subject by performing gait analysis. The SLS AFOs showed equivalence with a commercially available device over a number of gait parameters, including control of plantarflexion at toe off, a feature also seen in the gait patterns presented in the current article. It should be noted however that the AFO design used by Mavroidis et al. was very basic and did not have the same height as most currently prescribed AFOs due to the available build volume in the SLS machine used.

Schrank & Stanhope [13] tested the dimensional accuracy of the SLS process by building half scale AFOs at different orientations. They found the produced devices to have no dimensional discrepancies compared to the CAD model that were above 1.5mm, with the majority these discrepancies below 0.5mm. The authors also fabricated two full scale customised devices for two healthy adults and reported no adverse affects on gait and no discomfort after one hour, although it should be noted that no standardised or objective measures were used to report these outcomes.

Pallari et al. [11] have carried out, to the authors’ knowledge, the only existing study on a patient cohort, testing SLS fabricated FOs against standard, customised devices in a small group of participants with rheumatoid arthritis. The SLS devices demonstrated equivalence over the full set of outcome measures tested, including comfort and fit.

The applicability of AM for producing personalised sports footwear has also been investigated, with Salles & Gyi [12] producing simple “glove fit”, SLS fabricated insoles and measuring their effects on running performance and comfort in a running shoe against a shoe-only condition. No statistical differences in terms of performance between the two conditions were found due to the small number of subjects tested in this pilot study, however the feasibility of producing personalised sports insoles using AM was confirmed.

While the debate over off-the-shelf versus customised orthoses continues [22], the types of technological advances described in this article have been largely absent from the discussion. The design freedom realised by AM, perhaps combined with the latest advances in gait analysis, may have the potential to provide a number of new tools for clinicians to personalise orthotic devices. One of the intentions of this article is to encourage healthcare professionals involved in the prescription of orthotic devices for the foot and ankle to explore new ideas made possible by this technology.

### Obstacles

There are three main obstacles limiting the immediate exploitation of AM for FOs and AFOs. Firstly, while it is possible to produce CAD orthoses that require intricate and complex alterations to the shape and type, no single software package currently exists that would allow these to be made easily in a clinical setting. Secondly, in order to design a custom device, the CAD software requires a 3D scan of the anatomy of interest, either taken directly from the patient or from an impression cast. A number of commercial systems for foot scanning are now available [23], however anecdotal evidence from the authors’ experience suggests the primary barrier to the uptake of this approach is the restriction of the clinician’s ability to manipulate the foot and ankle position while it is being scanned.

Finally, current low cost (in terms of both materials and machine) AM systems are based on FDM technology, which does not have quite the same ability to create very intricate designs, primarily due to the lack of an inherent support material. The reduction in build time per device seen in SLS and SLA are also not possible with FDM, therefore it may only be suitable for low volume manufacturing. In addition, materials for SLS and SLA are significantly more expensive than those used by FDM machines. The costs estimates for the SLS devices manufactured for this study, particularly for the AFO, are still above those normally quoted for traditionally manufactured devices although the added value of the extra functionality that has been incorporated into the designs should be taken into account.

## Conclusions

The previously prohibitive costs and technological problems associated with AM continue to decrease towards levels where the technology may be a feasible proposition for the manufacture of custom and customised foot and ankle orthoses. The use of AM to fabricate standard designs of FOs and AFOs has been successfully demonstrated, with initial findings suggesting that these devices may show equivalence in terms of clinical performance, and this study presents preliminary evidence to demonstrate that biomechanical changes can be achieved using novel devices which take advantage of the design freedom provided by AM. Further research is however required to confirm that these changes translate into clinically relevant outcomes. Full integration with computer aided design and analysis software such as finite element or musculoskeletal modelling software may be required to fully exploit the technology and allow the devices to be further personalised to suit the patient.

## Competing interests

This article was produced as part of the work being carried out by the A-FOOTPRINT consortium, a group that incorporating a number of orthotic manufacturers including Firefly Orthoses (MM is founder and director of Firefly Orthoses) and Peacocks Medical Group (JP is research and development manager at Peacocks Medical Group). Although there are no plans to commercially produce the FO designs presented here at the moment, variations incorporating similar features may be developed in the future. JP is named as the inventor on two patents relating to novel orthotic devices which relate indirectly to the technologies discussed in this article.

## Authors’ contributions

ST developed the orthosis designs and performed the testing and analysis. JP and JW assisted in the development of the devices and their fabrication. JP, JM and KD contributed to the discussion of additive manufacturing technologies. MM assisted with the development of the devices and contributed to the discussion of the current state of the art. JP, KD and JW were involved in the conception of the research. All authors participated in critical revision of the manuscript and read and approved the final version.

## Pre-publication history

The pre-publication history for this paper can be accessed here:

http://www.biomedcentral.com/1471-2474/13/84/prepub
